# Segregation and potential functional impact of a rare stop-gain PABPC4L variant in familial atypical Parkinsonism

**DOI:** 10.1038/s41598-019-50102-6

**Published:** 2019-09-19

**Authors:** Muhammad Aslam, Anwar Ullah, Nagarajan Paramasivam, Nirosiya Kandasamy, Saima Naureen, Mazhar Badshah, Kafaitullah Khan, Muhammad Wajid, Rashda Abbasi, Roland Eils, Marc A. Brockmann, Matthias Schlesner, Nafees Ahmad, Jakob von Engelhardt

**Affiliations:** 1 Institute of Pathophysiology, University Medical Center of the Johannes Gutenberg University Mainz, Mainz, Germany; 2 Institute of Biomedical and Genetic Engineering (IBGE), Islamabad, Pakistan; 3 Department of Biochemistry, Quaid-i-Azam University, Islamabad, Pakistan; 4 Heidelberg Center for Personalized Oncology (DKFZ-HIPO), German Cancer Research Center (DKFZ), Heidelberg, Germany; 5 Department of Zoology, PMAS-Arid Agriculture University Rawalpindi, Rawalpindi, Pakistan; 6 Department of Neurology, Shaheed Zulfiqar Ali Bhutto Medical University, Islamabad, Pakistan; 7 Department of Zoology, University of Balochistan, Quetta, Pakistan; 8 Department of Biological sciences, University of Okara, Okara, Pakistan; 9 Center for Digital Health, Berlin Institute of Health and Charité Universitätsmedizin Berlin, Berlin, Germany; 10 Health Data Science Unit, Bioquant, Medical Faculty, University of Heidelberg, Heidelberg, Germany; 11 Department of Neurology, Neuroradiology section, University Medical Center of the Johannes Gutenberg University Mainz, Mainz, Germany; 12 Bioinformatics and Omics Data Analytics, German Cancer Research Center (DKFZ), Heidelberg, Germany

**Keywords:** Genetics of the nervous system, Medical genetics

## Abstract

Atypical parkinsonian disorders (APDs) comprise a group of neurodegenerative diseases with heterogeneous clinical and pathological features. Most APDs are sporadic, but rare familial forms have also been reported. Epidemiological and post-mortem studies associated APDs with oxidative stress and cellular protein aggregates. Identifying molecular mechanisms that translate stress into toxic protein aggregation and neurodegeneration in APDs is an active area of research. Recently, ribonucleic acid (RNA) stress granule (SG) pathways were discussed to be pathogenically relevant in several neurodegenerative disorders including APDs. Using whole genome sequencing, mRNA expression analysis, transfection assays and cell imaging, we investigated the genetic and molecular basis of a familial neurodegenerative atypical parkinsonian disorder. We investigated a family with six living members in two generations exhibiting clinical symptoms consistent with atypical parkinsonism. Two affected family members suffered from parkinsonism that was associated with ataxia. Magnetic resonance imaging (MRI) of these patients showed brainstem and cerebellar atrophy. Whole genome sequencing identified a heterozygous stop-gain variant (c.C811T; p.R271X) in the Poly(A) binding protein, cytoplasmic 4-like (*PABPC4L)* gene, which co-segregated with the disease in the family. *In situ* hybridization showed that the murine *pabpc4l* is expressed in several brain regions and in particular in the cerebellum and brainstem. To determine the functional impact of the stop-gain variant in the *PABPC4L* gene, we investigated the subcellular localization of *PABPC4L* in heterologous cells. Wild-type PABPC4L protein localized predominantly to the cell nucleus, in contrast to the truncated protein encoded by the stop-gain variant p.R271X, which was found homogeneously throughout the cell. Interestingly, the wild-type, but not the truncated protein localized to RasGAP SH3 domain Binding Protein (G3BP)-labeled cytoplasmic granules in response to oxidative stress induction. This suggests that the *PABPC4L* variant alters intracellular distribution and possibly the stress granule associated function of the protein, which may underlie APD in this family. In conclusion, we present genetic and molecular evidence supporting the role of a stop-gain PABPC4L variant in a rare familial APD. Our data shows that the variant results in cellular mislocalization and inability of the protein to associate with stress granules.

## Introduction

Atypical parkinsonian disorders (APDs) comprise a heterogeneous group of neurodegenerative diseases that share clinical features with idiopathic Parkinson’s disease, but generally do not respond well to antiparkinsonian medications^1^. Most cases of APDs are sporadic but rare familial forms have also been reported^2,3^. Epidemiological studies have previously associated stressors such as environmental toxins and oxidative stress with the etiology of APDs^4–6^. Post-mortem studies have grouped APDs as protein accumulation disorders (e.g. proteinopathies) based on cellular protein aggregates^7–9^. Notably, the molecular and cellular processes that translate stress into protein aggregation and neurodegeneration in most APDs remain poorly understood.

Stress granules (SGs) are cytoplasmic foci formed in response to cellular stress mainly to minimize stress-related RNA damage and to promotes cell survival^10^. Recently, several reports suggested that defective stress granules play a pathogenic role in several proteinopathies^11–16^. Observations such as elevated levels of oxidized RNA products in the cerebrospinal fluid and protein aggregates colocalizing with stress granule markers in some APDs indirectly support their involvement^6,14,15,17^. In this study, we investigated the genetic basis of a familial APD segregating as autosomal dominant condition and found a rare stop-gain variant in the stress granule associated protein PABPC4L as an underlying cause.

## Materials and Methods

### Study participants

**Figure 1:**
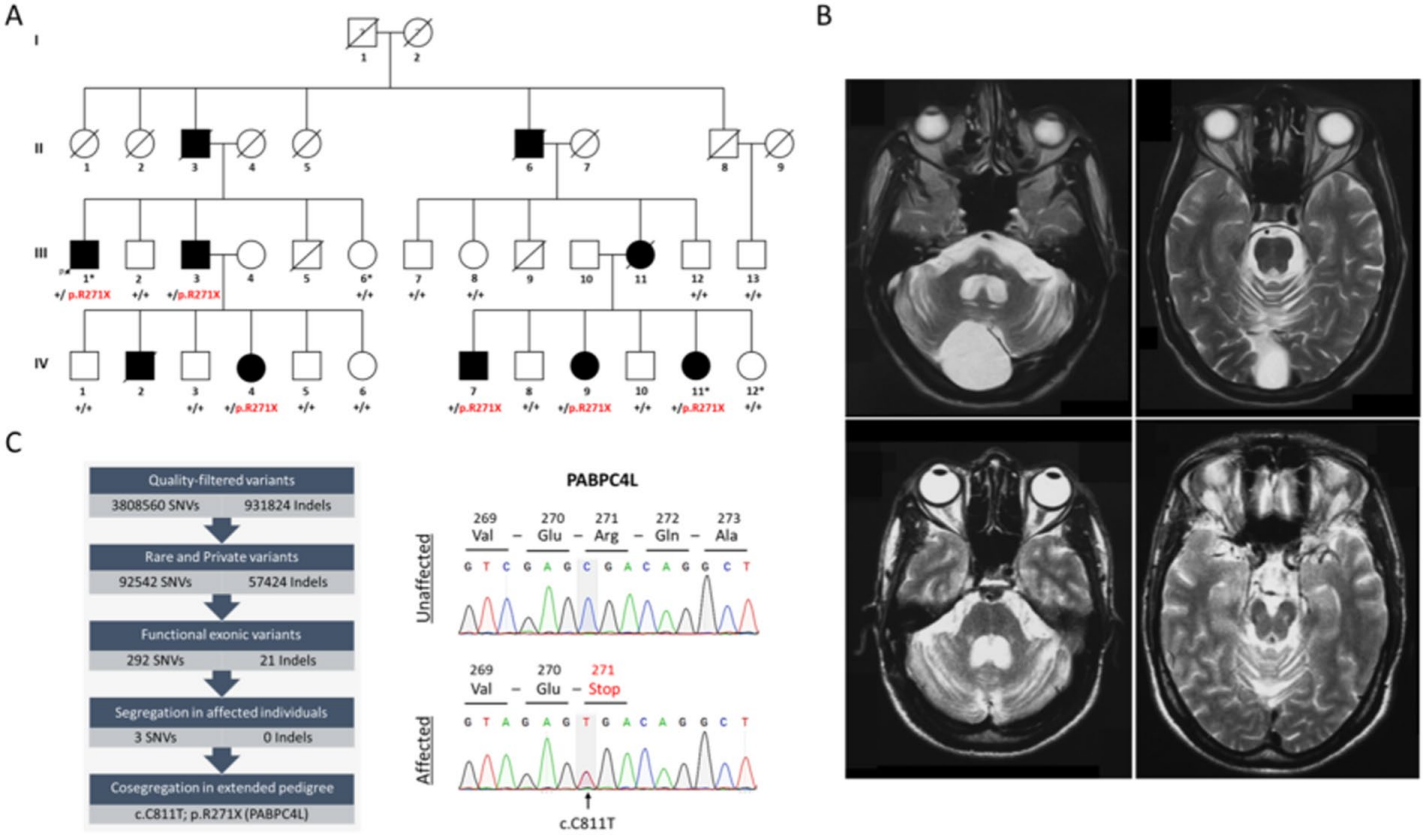
Clinical characteristics and genetic analysis of the family. (**A**) Pedigree of the family showing co-segregation of the stop-gain variant p.R271X in PABPC4L with atypical Parkinsonism. Squares represent males, and circles females. Shaded symbols indicate individuals that received a clinical diagnosis (see Table 1 for details of clinical information). The arrow indicates index patient. Individuals selected for WGS are marked with an asterisk. Deceased individuals are marked with diagonal lines. The family members provided retrospective information of affection status of the deceased individuals (II:3, II:6, III:11 and IV:2). Affection status of individuals in the first generation (I:1 and I:2) could not be ascertained. Question marks in the first generation indicate unknown affection status. Genotypes: +/+, homozygous for wild-type allele; and +/p.R271X, heterozygous. (**B**) MRI of brain of patient III:1 (top panel) and IV:7 (bottom panel). Both patients show comparable cerebellar atrophy with dilation of the fourth ventricle. In the patient III:1 (top panel) a retrocerebellar arachnoid cyst can be seen as an incidental finding, whereas the atrophy of the cerebellum is obvious. (**C**) Left panel: Analysis pipeline used in the identification of the candidate variants. Right panel: Representative Sanger sequencing chromatograms of an unaffected and affected family member illustrating the stop-gain variant c.C811T; p.271X in the PABPC4L. Grey highlighting indicates the variant position. Predicted consequence at the translational level is also shown. Sanger sequencing of additional affected and unaffected family members showed that the c.C811T; p.271X variant in PABPC4L segregated with the disease (Supplementary File 1).

Research procedures were performed in accordance with the Declaration of Helsinki. Approval of the study was obtained from the Institutional Review Board of the Institute of Biomedical and Genetic Engineering Islamabad. Written informed consent was obtained from all participants. A family with six members (Fig. 1A; III:1, III:3, IV:4, IV:7, IV:9 and IV:11) exhibiting a progressive neurodegenerative disease manifesting primarily as atypical parkinsonism was identified by M.B. who diagnosed and treated the patients at Pakistan Institute of Medical Sciences Islamabad. The MRI of patients with ataxia (III:1 and IV:7) were examined by M.A.B. A team of neurologists headed by M.B. examined the patients and provided the clinical records.

### Whole genome sequencing and quality control

Whole genome sequencing was performed for two affected (III:1, IV:11) and two unaffected family members (III:6, IV:12). Genomic DNA was isolated from peripheral blood cells using standard methods^18^. Libraries were prepared using the TruSeq Nano DNA PCR-free kit (Illumina San Diego CA USA). Paired-end sequencing (2 × 150 bp) was performed at >30× coverage per sample on Illumina HiSeqX TEN (Illumina San Diego CA). Raw reads were aligned to the human reference genome (version build GRCh37, version hs37d5) using bwa mem (version 0.7.8)^19^ with minimum base quality threshold set to zero [-T 0] and remaining settings left at default values. Duplicates were marked using Picard version (1.125)^20^. Single nucleotide variants (SNVs) were called using SAMtools (version 0.1.19)^21^ and short indels were called using Platypus (version 0.7.4)^22^. Functional classifications of the variants were done using ANNOVAR^23^ with gene model definitions from Gencode (version v19).

### Variant filtering and selection

Variants with base quality Q score <20, minimum coverage <10X, or a minor allele frequency MAF >0.001 in ExAC (version 0.3) or MAF >0.01 in the 1000 genomes were removed. Thereafter all functional mutations including: nonsynonymous SNVs; frameshift and non -frameshift indels; and stop-gain or stop-loss or splice-site SNVs or Indels were carried forward and were evaluated for deleteriousness using CADD [24] and a set of prediction tools including SIFT, PolyPhen2, LRT, Mutation Taster, FATHMM, PROVEAN, MetaSVM and MetaLR [25–31]. The WGS variants complying with the autosomal dominant mode of inheritance and segregating in the patients and not in controls were prioritized for Sanger sequencing validation and co-segregation testing in the extended pedigree.

### Sanger validation and co-segregation analysis

The genomic regions harboring prioritized variants were amplified using predesigned commercially obtained M13-tailed primer pairs [Primer ID (Gene_ID) Hs00253986_CE (*PABPC4L*), Hs00278134_CE (*ZNF292*), Hs00278167_CE (C6orf163), Thermo Fischer Scientific Inc.]. Thermocycling conditions used were as follows: 3 min at 94 °C, 30 cycles of 94 °C for 30 s, 60 °C for 30 s, and 72 °C for 45 s, and the final extension for 5 min at 72 °C. The PCR products were resolved on 1.5% agarose gel and amplified genomic fragments were purified using MinElute Gel Extraction Kit (Qiagen, Germany). Bi-directional Sanger sequencing was performed with M13 forward (TGTAAAACGACGGCCAGT) and reverse (CAGGAAACAGCTATGACC) primers (StarSEQ GmBH, Mainz, Germany).

### Plasmid preparation

Full length cDNA of wild-type human *PABPC4L* (NCBI Reference Sequence: NM_001114734.2) was amplified from human adult brain cDNA using forward primer containing XhoI site:5′-CACCCTCGAGACATGAATGTAGCAGCCAAGTACC-3′ and reverse primer containing XmaI site5′-ATATCCCGGGCTAGTGTCTCTGGGCCAAGG-3′ (PABPC4L specific sequence is underlined). PCR product was digested with XhoI and XmaI and inserted into pmCherry-C1 vector (Clontech). *PABPC4L* cDNA clone carrying the c.C811T variant encoding p.R271X was generated using Phusion Site-Directed Mutagenesis Kit (Thermo Fischer Scientific Inc.) and mutagenic primer AGAAAGTCGAGTGACAGGCTGAG (variant position is underlined) according to manufacturer’s instructions. Flag-tagged PABPC4L constructs was generated with PCR using forward primer containing the FLAG tag encoding sequence GACTACAAAGACGATGACGACAAG containing HindIII site. Construct encoding G3BP1 (peGFP-C1-G3BP1) was kindly provided by Nancy Kedersha (Brigham and Women’s Hospital, Boston, MA)^24^. Plasmids were purified with GeneJET Plasmid Midiprep Kit (Thermo Fischer Scientific Inc.). Clones generated by PCR were confirmed by direct sequencing.

### Cell culture and transfection

Human embryonic kidney 293 (HEK293) cells were cultured in Dulbecco’s Modified Eagle Medium (DMEM) supplemented with 10% fetal bovine serum and 1% penicillin–streptomycin (all from Gibco, Paisley, UK). Cells were cultured at 37 °C in a humidified atmosphere with 5% CO_2_. The medium was changed every 2–3 days. Cell cultures were tested for mycoplasma contamination prior to experiments using PCR Mycoplasma Test Kit I/C (PK-CA91-1024, PromoCell GmbH, Germany). For transient transfections, cells were plated either in 12 well plates or on glass covers slips coated with 200 μl of 0.01% poly-L-Lysine solution (sigma). Transfections were performed using Lipofectamine 2000 reagent (Invitrogen, Carlsbad, CA, USA). For induction of oxidative stress, cell culture medium was replaced with fresh complete Dulbecco’s Modified Eagle’s Medium (DMEM) growth media one hour prior to stress induction. Cells were stressed with sodium arsenite (0.5 mM, Sigma-Aldrich) for 45 min at 37 °C in an incubator with 5% CO_2_.

### Western blotting

HEK293 cells were washed twice with phosphate buffer saline (PBS) and lysed thirty-six hours post-transfection. Western blotting was performed with cell lysates containing 30 μg of proteins. After sodium dodecyl sulfate polyacrylamide gel electrophoresis, the proteins were transferred to polyvinylidene difluoride (PVDF) membrane, which was blocked in 10% skimmed milk for one hour. The membranes were then incubated with mouse anti-Flag (Sigma) overnight at 4 °C, followed by incubation with peroxidase-linked secondary antibodies (1:10,000) for one hour. Bands were detected on a ChemiDoc XRS+ imaging system (Bio-Rad). After Flag tag detection, PVDF membrane was stripped, and then subjected to detect beta-Actin expression using Rabbit anti-Actin antibody (1:1000, Abcam).

### Cell imaging,quantification and analysis

HEK293 cells grown and transfected on glass coverslips were washed three times with phosphate buffer solution (PBS) and were fixed in 4% paraformaldehyde for 10 minutes. Cells were washed again three times with PBS and nuclei were stained with 4′, 6′-diamidino-2-phenylindole (DAPI; Sigma, St Louis, MO, USA). Coverslips were then mounted onto slides with ProLong Gold *Antifade* Mountant (Thermo Fischer Scientific Inc.). Images were obtained with a 63x objective on a TCS SP5 confocal imaging system (Leica Microsystems, Heidelberg GmbH). The PABPC4L localization was quantified by calculating a nuclear to cytoplasmic ratio using Fiji software as described previously^25,26^. Briefly, cells were imaged for mCherry (expressed as a fusion protein with wild-type or PABPC4L:p.R271X variant) and DAPI fluorescence. The nuclear area was identified by DAPI and cytoplasmic area as total mCherry signal minus nuclear area. We determined the integrated fluorescence intensity ratio of nuclear/cytoplasmic mCherry signal. Twenty-four cells for each condition from triplicate coverslips were processed and ratios were plotted using QtiPlot software. Stress granules (SGs) were identified by green fluorescent protein tagged RasGAP SH3 domain Binding Protein (G3BP-GFP) expression as described previously^27^. Only cells with at least three G3BP-GFP positive foci with a size of 0.5–2.5 μm were included. Fiji software was used to quantify the integrated intensity of mCherry fluorescence (i.e PABPC4L) overlapping with GFP signal (i.e G3BP). At least thirty cells for each condition from triplicate coverslips were analyzed.

### *In situ* hybridization

Animal care and experimental procedures were performed in agreement with the German law on the use of laboratory animals (animal welfare act; TierSchG). All procedures involving mice were performed according to the protocols approved by the German Cancer Research Center institutional animal care and use committee and by the local responsible government department (Regierungspräsidium Karlsruhe). Brains from embryonic, developing and adult C57Bl/6 mice were frozen on dry ice and 15 μm sections were cut on a cryostat (Leica Microsystems, Germany). *In situ* hybridization experiments were carried out as described^28^ with the radiolabeled oligodeoxyribonucleotide probes targeting the exon-exon junction region of the mouse *pabpc4l* mRNA transcript (NM_001101479):

[5′-CTGCAGTCCATAACCTGGAAGCTACAGCCTTGAGAGCTGCAGGCTTGTA-3′].

The probe was 3′ end-labeled with (a)-^33^P-dATP (Hartmann Analytic, Germany) using terminal deoxynucleotidetransferase (*Roche*, Basel, Switzerland). Brain sections were then incubated overnight in ahybridization mix containing 4 × SSC (0.6 M NaCl, 0.06 M sodium citrate), 50% formamide, 10% dextrane and 1 pg/μl labeled oligodeoxyribonucleotide probe at 42 °C and were subsequently washed three times at 56 °C for 30 min. Brain sections were then dehydrated and exposed to Kodak R X-omat AR film for 1 week. Control *in situ* hybridization was performed by adding excess amount (10x) of cold competitor (unlabeled probe) to the hybridization mix.

### Statistical analysis

Data were plotted as mean and SD using QtiPlot. Shapiro-Wilk-test and Student’s t-test were used for assessing normality of data and to compare means, respectively. P-values of less than 0.05 were considered statistically significant.

## Results

The index patient III:1 (Fig. 1A, arrowhead), 47-year-old male, presented with two years history of walking difficulty, trouble in dressing and writing and problems with right hand coordination. On the neurological examination, spasticity on his left side and gait ataxia were noticed. His tendon reflexes in lower limbs were brisk and plantar responses were bilateral absent. During the next two years, he developed Parkinson-like symptoms comprising rigidity and bradykinesia as well as progression of his ataxia and downbeat nystagmus. In addition, his voice weakened, he suffered of dysarthria and dysautonomia with constipation and nocturia. His cognitive abilities were intact (MMSE score 28/30). Neuroimaging studies showed an atrophy of cerebellum, middle cerebellar peduncles and brainstem (Fig. 1B, top panel). There were no abnormalities in striatum, globus pallidus, corpus callosum and thalamus. Cerebrospinal fluid and serological investigations with a complete thyroid panel and B12 level were unremarkable. Patient III:3 (Fig. 1A), brother of index patient III:1, was diagnosed with parkinsonism, dysautonomia and insomnia at slightly younger age (42 years). He did not exhibit ataxia or tremor at the time of his diagnosis. His daughter (IV:4; Fig. 1A) exhibited unilateral tremor and rigidity in her right arm at the age of thirty-four. Patient IV:7(male) was first noticed at an age of 38 years with ataxic gait during his training as a soldier. He developed parkinsonian symptoms similar to patient III:1 with additional intention tremor and depression. He died during the course of this study at the age of 45 apparently due to cardiopulmonary arrest. His family members retrospectively informed us that he had developed difficulty breathing during sleep, forcing him to sleep upright to avoid dyspnea. Neuroimaging of IV:7 showed cerebellar atrophy with dilation of the fourth ventricle (Fig. 1A,B bottom panel). Two sisters of the patient IV:7 (Fig. 1A, IV:9 and IV:11) exhibited parkinsonism at the age of 37 and 35, respectively. Patients described here received levodopa for their parkinsonian symptoms, which did not improve symptoms even at high doses. Demographic and clinical description of all cases is summarized in Table 1.

**Table 1:** Clinical characteristics of the affected family members.

Sex	III:1	III:3	IV:4	IV:7	IV:9	IV:11
	Male	Male	Female	Male	Female	Female
Age at onset, year	47	42	34	38	37	35
Current age, year	55	65	38	47	43	40
Initial Symptoms	Ataxia	Akinesia	Tremor	Ataxia	Tremor	Tremor
**Neurologic findings**						
Akinesia	+	+	+	+	+	+
Spasticity	+	−	−	+	−	−
Rigidity	+	+	+	+	+	+
Deep tendon reflex	Brisk	Normal	Normal	Brisk	Normal	Normal
Ataxia	+	−	−	+	−	−
Tremor	−	−	+	+	+	+
**Other Symptoms**						
Autonomic disturbance	+	+	−	+	+	−
Depression	−	−	−	+	−	−
Sleepdisorder	−	+	−	+	−	−

Family-based analyses of the WGS data obtained for two affected and two unaffected members (asterisks in Fig. 1A) assuming an autosomal dominant mode of inheritance prioritized heterozygous variants in PABPC4L, ZNF292 and C6orf163 for sanger validation and disease co-segregation testing in the extended pedigree (Fig. 1C; left panel and Supplementary Table). Sanger sequencing of the three prioritized variants in four additional affected (Fig. 1A, III:3, IV:4, IV:7 and IV:9) and eleven unaffected biological relatives excluded ZNF292 and C6orf163 variants and identified the stop-gain variant c.C811T; p.R271X in the *PABPC4L* gene as the possible cause of parkinsonian disorder in the family (Supplementary Table). The c.C811T;p.R271X variant in *PABPC4L* is absent in the south Asian control cohort in the GnomAd^29^ and in-house datasets. This variant was ranked among the top 0.1% most deleterious possible substitutions (CADD score 38).

**Figure 2:**
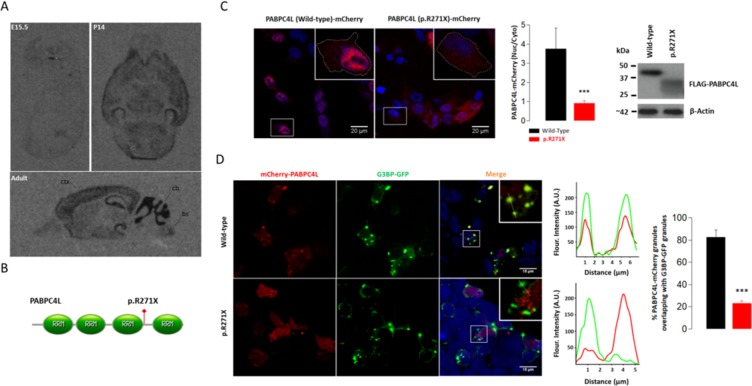
Expression, subcellular localization and function of PABPC4L. (**A**) Distribution of *pabpc4l* mRNA in the developing and adult mouse brain. Sections were examined for *pabpc4l* mRNA expression at embryonic day 15.5 (E15.5), postnatal day 14 (P14) or at adult stage (ctx; cortex, cb; cerebellum, bs; brainstem). (**B**) Predicted structure of the PABPC4L protein. RRM; RNA recognition motifs. The variant position is indicated in red. (**C**) Left panel: Cellular distribution of mCherry-tagged wild-type and p.R271X variant containing PABPC4L in HEK293 cells. Wild-type PABPC4L-mCherry shows a nuclear distribution, whereas PABPC4L (p.R271X)-mCherry shows a uniform nucleo-cytoplasmic distribution in HEK293 cells. Red: mCherry fluorescence, Blue: DAPI nuclei. The panel on the right shows the nuclear to cytoplasmic ratio of the mCherry signal in HEK293 cells transfected with either wild-type PABPC4L-mCherry or p.R271X PABPC4L-mCherry plasmids. Right panel: Western blot analysis of FLAG-tagged wild-type and p.R271X variant containing PABPC4L in HEK293 cells. The p.R271X variant reduces the molecular weight of PABPC4L. (**D**) Wild type but not p.R271X variant PABPC4L localizes to G3BP-GFP-positive stress granules in Na-arsenite treated HEK293 cells. Red: PABPC4L-mCherry, green: G3BP-GFP. A region including G3BP-GFP positive foci is enlarged. The graph indicates the line scan analysis of the cytoplasmic region (green line: G3BP-GFP signal, red line: PABPC4L-mCherry signal) and shows the extent of colocalization. The bar graph on the right panel shows the quantification of red signal intensity overlapping with G3BP-GFP signal. t-test, Means ± S.D. ***p = 0.0001.

PABPC4L gene is previously uncharacterized. Thus, we examined the spatiotemporal expression pattern of *pabpc4l* in the mouse brain. The level of *pabpc4l* transcript was low at embryonic day 15 (Fig. 2A; E15.5). There was a developmental upregulation of the *pabpc4l* transcript in the cerebellum with moderate signal intensity at P14 (Fig. 2A; P14) and strong intensity in the adult brain. The *pabpc4l* transcript was also detected in cortex, hippocampus, presubiculum, and regions of the brainstem of adult mice (Fig. 2A; Adult). In the adult human brain PABPC4L transcript was detected at higher levels in the hindbrain particularly in cerebellum whereas moderate expression was also detected in regions of midbrain and forebrain (Supplementary Fig. 2, credit: Human microarray data from the Allen Brain Atlas)^30^.

The human *PABPC4L* transcript encodes a 370 amino acids protein (UniProt: P0CB38) predicted to contain four non-identical RNA recognition motifs (RRMs) (RRM1: 10–88, RRM2: 98–174, RRM3: 190–267 and RRM4: 293–369) characteristic of cytoplasmic type I polyadenylate-binding proteins (PABPs) i.e PABPC1, PABPC4 and PABPC5^31^. The identified stop-gain variant p.R271X results in a shorter PABP4CL protein lacking the C-terminal RRM motif (RRM4: 293–369).

To investigate functional consequences of the PABPC4L variant p.R271X, we expressed wild-type and the truncated PABPC4L in HEK293 cells as mCherry or flag-tag fusion proteins. Since RRM motifs influence the subcellular localization of PABP proteins^32^, we examined the subcellular localization of the wild-type and p.R271X variant PABPC4L proteins in HEK293 cells. Wild-type PABPC4L-mCherry fusion protein showed a predominantly nuclear localization in HEK293 cells. In stark contrast, the truncated PABPC4L variant exhibited a uniform nucleo-cytoplasmic localization with no aggregation in the nucleus, suggesting that the stop-gain variant impairs nuclear import or retention (Fig. 2C, Left panel).Detection of the truncated protein 48-hours post transfection at comparable levels with wild-type PABPC4L protein suggests that the variant does not result in increased degradation of the protein (Fig. 2C, right panel).

Cytoplasmic PABPs PABPC1 and PABPC4 have recently been identified to associate with RasGAP SH3 domain Binding Protein (G3BP) labeled stress granules^33,34^. To test similar association of PABPC4L with RNA stress granules we exposed PABPC4L-mCherry transfected HEK293 cells to sodium arsenite induced oxidative stress and found a redistribution of wild-type PABPC4L from a predominantly nuclear to a focal cytoplasmic pattern. Moreover, PABPC4L-mCherry co-localized with the stress granule marker G3BP-GFP, indicating that PABPC4L associates with stress granules (Fig. 2D). The truncated protein PABPC4L(p.R271X)-mCherry also formed cytoplasmic foci in some cells, but showed significantly less co-localization with G3BP-GFP, suggesting that the p.R271X variant alters the function of the protein (Fig. 2D).

## Discussion

We identified a rare variant (c.C811T; p.R271X) in PABPC4L segregating in a family with atypical parkinsonian disease (Fig. 1). Parkinsonism in the family shows peculiar features e.g. rapidly progressing heterogeneous Levodopa unresponsive motor disturbances, early autonomic dysfunction, and additional presence of ataxia, cerebellar atrophy and depression in some affected family members (Figure B and Table 1). Our data together with a previous report of an association of a genomic deletion affecting PABPC4L with treatment resistant depression^35^ indicate that genetic abnormalities at PABPC4L locus may cause detrimental changes in brain function. Collectively the data presented in this report extend the genetic and molecular landscape of APDs.

Mouse *pabpc4l* transcript is expressed in several brain areas including cerebellum, hippocampus, cortex and brainstem. The transcript is comparably low prenatally and shows postnatal upregulation with a particularly high expression in the cerebellum of adult mice. Given the regional gene expression in mouse and human brains is generally conserved^36^, the postnatal upregulation of cerebellar expression may explain the onset of symptoms in early to middle adulthood (onset between 34 to 47 years) and presentation of disease with cerebellar symptoms and cerebellar atrophy in PABPC4L (c.C811T; p.R271X) variant carriers. Furthermore in the human brain PABPC4L transcript was detected in several brain regions with motor and autonomic functions (Supplementary Fig. 2, image credit: Human microarray data from the Allen Brain Atlas^30^. Taken together the expression pattern of PABPC4L in brain support the involvement PABPC4L (c.C811T; p.R271X) variant in the clinical outcome observed in the family.

Molecular functions and cellular roles of PABPC4L are currently not known. We here show that wild-type PABPC4L redistributes from a predominantly nuclear to focal cytoplasmic pattern and co-localizes with G3BP upon stress induction (Fig. 2C,D). This suggests that PABPC4L may associate with stress granules similar to cytoplasmic PABP family members PABPC1 and PABPC4^33,34^. Unlike PABPC1 and PABPC4, PABPC4L has not been shown to bind poly(A) RNA as yet and PABPC4L lacks an extended carboxy-terminus shared by PABPC1 and PABPC4^37,38^. However, since the additional carboxy-terminal sequence is dispensible for viability in yeast or PABP function in Xenopus, PABPC4L may thus share stress granule associated function with PABPC1 and PABPC4^39,40^. Our observations also corroborate data from an interactome study, which reported PABPC4L as an interacting partner of the two core stress granule proteins CSDE1and PQBP1, both of which are indispensable for functional G3BP containing stress granules^41–43^. More importantly, the truncated PABPC4L protein due to the p.R271X variant shows altered subcellular localization in unstressed cells and diminished stress granule association upon stress induction which may result in functional haploinsufficiency of PABPC4L in the c.C811T; p.R271X carriers and thus may be the cause of disease in the patients.

Our study adds to the growing information supporting a role of stress granule associated proteins and RNA metabolism in neurodegenerative pathologies. Stress granules protect cellular RNAs from oxidative damage and promote selective translation of stress response proteins^10,44,45^. Formation of stress granules also protects cells from stress-induced apoptosis by promoting the activity of the antioxidant enzyme USP10^46,47^. Moreover, defective stress granules may promote protein aggregation and contribute to formation of intracellular inclusions^48^.The findings of this study should however be treated with caution due to the use of heterologous expression model such as HEK293. We selected HEK293 cells primarily because they express major stress granule proteins endogenously including PABP family members (https://www.proteinatlas.org) and stress granules can robustly be induced in HEK293 cells. Thus HEK293 cells are frequently used for investigating stress granule associated proteins^49–51^. Predominant exclusion of the truncated PABPC4L protein from stress granules upon induction of stress in HEK293 cells as observed in our study warrants further investigation in more sophisticated, tractable and accurate cellular disease models such as patient-derived iPSCs or neuronal cells to understand a link between neurodegeneration and altered cellular distribution of PABPC4L in p.R271X variant carriers.

## Conclusions

In conclusion, a truncating variant in RNA stress granule associated protein PABPC4L was identified as the potential cause for familial atypical parkinsonism. Our data indicate cytosolic mislocalization of truncated PABPC4L in unstressed and stressed cells. Future studies focusing on the role of stress granule recruitment of PABPC4L and its relevance to the cellular stress responses may unravel novel mechanism of neurodegeneration.

### Ethics approval and consent to participate

Ethics committee of the Institute for Biotechnology and Genetic Engineering (IBGE, Islamabad, Pakistan) provided the approval (Ref. IBGE/SARK04/1201/2012). Written informed consent was obtained from all participants.

### Consent for publication

All authors critically revised the manuscript and approved the final version before submission.

## Supplementary information

Supplementary file

## Data Availability

The datasets used and/or analysed during the current study are available from the corresponding author on reasonable request.

## References

[1] Stamelou Maria, Quinn Niall P., Bhatia Kailash P. (2013). “Atypical” atypical parkinsonism: New genetic conditions presenting with features of progressive supranuclear palsy, corticobasal degeneration, or multiple system atrophy-A diagnostic guide. Movement Disorders.

[2] Wullner U, Schmitt I, Kammal M, Kretzschmar H A, Neumann M (2008). Definite multiple system atrophy in a German family. Journal of Neurology, Neurosurgery & Psychiatry.

[3] Hara K (2007). Multiplex families with multiple system atrophy. Archives of neurology.

[4] Caparros-Lefebvre Dominique, Steele John (2005). Atypical parkinsonism on Guadeloupe, comparison with the parkinsonism–dementia complex of Guam, and environmental toxic hypotheses. Environmental Toxicology and Pharmacology.

[5] Chirichigno Jason W, Manfredi Giovanni, Beal M.Flint, Albers David S (2002). Stress-induced mitochondrial depolarization and oxidative damage in PSP cybrids. Brain Research.

[6] Kikuchi Akio, Takeda Atsushi, Onodera Hiroshi, Kimpara Teiko, Hisanaga Kinya, Sato Nobuyuki, Nunomura Akihiko, Castellani Rudy J., Perry George, Smith Mark A., Itoyama Yasuto (2002). Systemic Increase of Oxidative Nucleic Acid Damage in Parkinson's Disease and Multiple System Atrophy. Neurobiology of Disease.

[7] Dickson D. W. (2012). Parkinson's Disease and Parkinsonism: Neuropathology. Cold Spring Harbor Perspectives in Medicine.

[8] Wakabayashi Koichi, Takahashi Hitoshi (2004). Pathological heterogeneity in progressive supranuclear palsy and corticobasal degeneration. Neuropathology.

[9] Jellinger Kurt A. (2008). Neuropathological Aspects of Alzheimer Disease, Parkinson Disease and Frontotemporal Dementia. Neurodegenerative Diseases.

[10] Protter David S.W., Parker Roy (2016). Principles and Properties of Stress Granules. Trends in Cell Biology.

[11] McDonald KK (2011). TAR DNA-binding protein 43 (TDP-43) regulates stress granule dynamics via differential regulation of G3BP and TIA-1. Human Molecular Genetics.

[12] Maharjan Niran, Künzli Christina, Buthey Kilian, Saxena Smita (2016). C9ORF72 Regulates Stress Granule Formation and Its Deficiency Impairs Stress Granule Assembly, Hypersensitizing Cells to Stress. Molecular Neurobiology.

[13] Brunello, C. A., Yan, X. & Huttunen, H. J. Internalized Tau sensitizes cells to stress by promoting formation and stability of stress granules. *Scientific Reports*, 10.1038/srep30498 (2016). 10.1038/srep30498 PMC4962319 27460788

[14] Liu-Yesucevitz Liqun, Bilgutay Aylin, Zhang Yong-Jie, Vanderwyde Tara, Citro Allison, Mehta Tapan, Zaarur Nava, McKee Ann, Bowser Robert, Sherman Michael, Petrucelli Leonard, Wolozin Benjamin (2010). Tar DNA Binding Protein-43 (TDP-43) Associates with Stress Granules: Analysis of Cultured Cells and Pathological Brain Tissue. PLoS ONE.

[15] Dormann Dorothee, Haass Christian (2011). TDP-43 and FUS: a nuclear affair. Trends in Neurosciences.

[16] Thomas Matthew, Alegre-Abarrategui Javier, Wade-Martins Richard (2013). RNA dysfunction and aggrephagy at the centre of an amyotrophic lateral sclerosis/frontotemporal dementia disease continuum. Brain.

[17] Espay A. J., Spina S., Houghton D. J., Murrell J. R., de Courten-Myers G. M., Ghetti B., Litvan I. (2010). Rapidly progressive atypical parkinsonism associated with frontotemporal lobar degeneration and motor neuron disease. Journal of Neurology, Neurosurgery & Psychiatry.

[18] Sambrook, J., Fritsch, E. F. & Maniatis, T. Molecular Cloning: A Laboratory Manual. *Cold Spring Harbor laboratory press*. *New York* (1989).

[19] Li H, Durbin R (2010). Fast and accurate long-read alignment with Burrows-Wheeler transform. Bioinformatics.

[20] Tischler G, Leonard S (2014). biobambam: tools for read pair collation based algorithms on BAM files. Source Code for Biology and Medicine.

[21] Li H (2009). The Sequence Alignment/Map format and SAMtools. Bioinformatics.

[22] Rimmer A (2014). Integrating mapping-, assembly- and haplotype-based approaches for calling variants in clinical sequencing applications. Nature genetics.

[23] Wang K., Li M., Hakonarson H. (2010). ANNOVAR: functional annotation of genetic variants from high-throughput sequencing data. Nucleic Acids Research.

[24] Kedersha Nancy, Tisdale Sarah, Hickman Tyler, Anderson Paul (2008). Chapter 26 Real‐Time and Quantitative Imaging of Mammalian Stress Granules and Processing Bodies. Methods in Enzymology.

[25] Sociale Mariangela, Wulf Anna-Lena, Breiden Bernadette, Klee Kathrin, Thielisch Melanie, Eckardt Franka, Sellin Julia, Bülow Margret H., Löbbert Sinah, Weinstock Nadine, Voelzmann André, Schultze Joachim, Sandhoff Konrad, Bauer Reinhard (2018). Ceramide Synthase Schlank Is a Transcriptional Regulator Adapting Gene Expression to Energy Requirements. Cell Reports.

[26] Schindelin Johannes, Arganda-Carreras Ignacio, Frise Erwin, Kaynig Verena, Longair Mark, Pietzsch Tobias, Preibisch Stephan, Rueden Curtis, Saalfeld Stephan, Schmid Benjamin, Tinevez Jean-Yves, White Daniel James, Hartenstein Volker, Eliceiri Kevin, Tomancak Pavel, Cardona Albert (2012). Fiji: an open-source platform for biological-image analysis. Nature Methods.

[27] Kedersha Nancy, Stoecklin Georg, Ayodele Maranatha, Yacono Patrick, Lykke-Andersen Jens, Fritzler Marvin J., Scheuner Donalyn, Kaufman Randal J., Golan David E., Anderson Paul (2005). Stress granules and processing bodies are dynamically linked sites of mRNP remodeling. The Journal of Cell Biology.

[28] Farrow, P. *et al*. Auxiliary subunits of the CKAMP family differentially modulate AMPA receptor properties. *eLife* 4 (2015). 10.7554/eLife.09693 PMC4733035 26623514

[29] Lek Monkol, Karczewski Konrad J., Minikel Eric V., Samocha Kaitlin E., Banks Eric, Fennell Timothy, O’Donnell-Luria Anne H., Ware James S., Hill Andrew J., Cummings Beryl B., Tukiainen Taru, Birnbaum Daniel P., Kosmicki Jack A., Duncan Laramie E., Estrada Karol, Zhao Fengmei, Zou James, Pierce-Hoffman Emma, Berghout Joanne, Cooper David N., Deflaux Nicole, DePristo Mark, Do Ron, Flannick Jason, Fromer Menachem, Gauthier Laura, Goldstein Jackie, Gupta Namrata, Howrigan Daniel, Kiezun Adam, Kurki Mitja I., Moonshine Ami Levy, Natarajan Pradeep, Orozco Lorena, Peloso Gina M., Poplin Ryan, Rivas Manuel A., Ruano-Rubio Valentin, Rose Samuel A., Ruderfer Douglas M., Shakir Khalid, Stenson Peter D., Stevens Christine, Thomas Brett P., Tiao Grace, Tusie-Luna Maria T., Weisburd Ben, Won Hong-Hee, Yu Dongmei, Altshuler David M., Ardissino Diego, Boehnke Michael, Danesh John, Donnelly Stacey, Elosua Roberto, Florez Jose C., Gabriel Stacey B., Getz Gad, Glatt Stephen J., Hultman Christina M., Kathiresan Sekar, Laakso Markku, McCarroll Steven, McCarthy Mark I., McGovern Dermot, McPherson Ruth, Neale Benjamin M., Palotie Aarno, Purcell Shaun M., Saleheen Danish, Scharf Jeremiah M., Sklar Pamela, Sullivan Patrick F., Tuomilehto Jaakko, Tsuang Ming T., Watkins Hugh C., Wilson James G., Daly Mark J., MacArthur Daniel G. (2016). Analysis of protein-coding genetic variation in 60,706 humans. Nature.

[30] Hawrylycz Michael J., Lein Ed S., Guillozet-Bongaarts Angela L., Shen Elaine H., Ng Lydia, Miller Jeremy A., van de Lagemaat Louie N., Smith Kimberly A., Ebbert Amanda, Riley Zackery L., Abajian Chris, Beckmann Christian F., Bernard Amy, Bertagnolli Darren, Boe Andrew F., Cartagena Preston M., Chakravarty M. Mallar, Chapin Mike, Chong Jimmy, Dalley Rachel A., Daly Barry David, Dang Chinh, Datta Suvro, Dee Nick, Dolbeare Tim A., Faber Vance, Feng David, Fowler David R., Goldy Jeff, Gregor Benjamin W., Haradon Zeb, Haynor David R., Hohmann John G., Horvath Steve, Howard Robert E., Jeromin Andreas, Jochim Jayson M., Kinnunen Marty, Lau Christopher, Lazarz Evan T., Lee Changkyu, Lemon Tracy A., Li Ling, Li Yang, Morris John A., Overly Caroline C., Parker Patrick D., Parry Sheana E., Reding Melissa, Royall Joshua J., Schulkin Jay, Sequeira Pedro Adolfo, Slaughterbeck Clifford R., Smith Simon C., Sodt Andy J., Sunkin Susan M., Swanson Beryl E., Vawter Marquis P., Williams Derric, Wohnoutka Paul, Zielke H. Ronald, Geschwind Daniel H., Hof Patrick R., Smith Stephen M., Koch Christof, Grant Seth G. N., Jones Allan R. (2012). An anatomically comprehensive atlas of the adult human brain transcriptome. Nature.

[31] Eliseeva I. A., Lyabin D. N., Ovchinnikov L. P. (2013). Poly(A)-binding proteins: Structure, domain organization, and activity regulation. Biochemistry (Moscow).

[32] Gray N. K., Hrabalkova L., Scanlon J. P., Smith R. W. P. (2015). Poly(A)-binding proteins and mRNA localization: who rules the roost?. Biochemical Society Transactions.

[33] Markmiller Sebastian, Soltanieh Sahar, Server Kari L., Mak Raymond, Jin Wenhao, Fang Mark Y., Luo En-Ching, Krach Florian, Yang Dejun, Sen Anindya, Fulzele Amit, Wozniak Jacob M., Gonzalez David J., Kankel Mark W., Gao Fen-Biao, Bennett Eric J., Lécuyer Eric, Yeo Gene W. (2018). Context-Dependent and Disease-Specific Diversity in Protein Interactions within Stress Granules. Cell.

[34] Burgess H. M., Richardson W. A., Anderson R. C., Salaun C., Graham S. V., Gray N. K. (2011). Nuclear relocalisation of cytoplasmic poly(A)-binding proteins PABP1 and PABP4 in response to UV irradiation reveals mRNA-dependent export of metazoan PABPs. Journal of Cell Science.

[35] O’Dushlaine Colm, Ripke Stephan, Ruderfer Douglas M., Hamilton Steven P., Fava Maurizio, Iosifescu Dan V., Kohane Isaac S., Churchill Susanne E., Castro Victor M., Clements Caitlin C., Blumenthal Sarah R., Murphy Shawn N., Smoller Jordan W., Perlis Roy H. (2014). Rare Copy Number Variation in Treatment-Resistant Major Depressive Disorder. Biological Psychiatry.

[36] Strand Andrew D., Aragaki Aaron K., Baquet Zachary C., Hodges Angela, Cunningham Philip, Holmans Peter, Jones Kevin R., Jones Lesley, Kooperberg Charles, Olson James M. (2007). Conservation of Regional Gene Expression in Mouse and Human Brain. PLoS Genetics.

[37] Tsai Yihsuan S., Gomez Shawn M., Wang Zefeng (2014). Prevalent RNA recognition motif duplication in the human genome. RNA.

[38] Mangus David A, Evans Matthew C, Jacobson Allan (2003). Genome Biology.

[39] Sachs A B, Davis R W, Kornberg R D (1987). A single domain of yeast poly(A)-binding protein is necessary and sufficient for RNA binding and cell viability. Molecular and Cellular Biology.

[40] Kühn Uwe, Pieler Tomas (1996). XenopusPoly(A) Binding Protein: Functional Domains in RNA Binding and Protein – Protein Interaction. Journal of Molecular Biology.

[41] Hein Marco Y., Hubner Nina C., Poser Ina, Cox Jürgen, Nagaraj Nagarjuna, Toyoda Yusuke, Gak Igor A., Weisswange Ina, Mansfeld Jörg, Buchholz Frank, Hyman Anthony A., Mann Matthias (2015). A Human Interactome in Three Quantitative Dimensions Organized by Stoichiometries and Abundances. Cell.

[42] Youn Ji-Young, Dunham Wade H., Hong Seo Jung, Knight James D.R., Bashkurov Mikhail, Chen Ginny I., Bagci Halil, Rathod Bhavisha, MacLeod Graham, Eng Simon W.M., Angers Stéphane, Morris Quaid, Fabian Marc, Côté Jean-François, Gingras Anne-Claude (2018). High-Density Proximity Mapping Reveals the Subcellular Organization of mRNA-Associated Granules and Bodies. Molecular Cell.

[43] Kunde S. A., Musante L., Grimme A., Fischer U., Muller E., Wanker E. E., Kalscheuer V. M. (2011). The X-chromosome-linked intellectual disability protein PQBP1 is a component of neuronal RNA granules and regulates the appearance of stress granules. Human Molecular Genetics.

[44] Wolozin B (2012). Regulated protein aggregation: stress granules and neurodegeneration. Molecular neurodegeneration.

[45] Ross Buchan J (2014). MRNP granules Assembly, function, and connections with disease. RNA Biology.

[46] Soncini Chiara, Berdo Ingrid, Draetta Giulio (2001). Ras–GAP SH3 domain binding protein (G3BP) is a modulator of USP10, a novel human ubiquitin specific protease. Oncogene.

[47] Takahashi M., Higuchi M., Matsuki H., Yoshita M., Ohsawa T., Oie M., Fujii M. (2012). Stress Granules Inhibit Apoptosis by Reducing Reactive Oxygen Species Production. Molecular and Cellular Biology.

[48] Wolozin Benjamin (2012). Regulated protein aggregation: stress granules and neurodegeneration. Molecular Neurodegeneration.

[49] Souquere S., Mollet S., Kress M., Dautry F., Pierron G., Weil D. (2009). Unravelling the ultrastructure of stress granules and associated P-bodies in human cells. Journal of Cell Science.

[50] Uhlen M., Fagerberg L., Hallstrom B. M., Lindskog C., Oksvold P., Mardinoglu A., Sivertsson A., Kampf C., Sjostedt E., Asplund A., Olsson I., Edlund K., Lundberg E., Navani S., Szigyarto C. A.-K., Odeberg J., Djureinovic D., Takanen J. O., Hober S., Alm T., Edqvist P.-H., Berling H., Tegel H., Mulder J., Rockberg J., Nilsson P., Schwenk J. M., Hamsten M., von Feilitzen K., Forsberg M., Persson L., Johansson F., Zwahlen M., von Heijne G., Nielsen J., Ponten F. (2015). Tissue-based map of the human proteome. Science.

[51] Bosco Daryl A., Lemay Nathan, Ko Hae Kyung, Zhou Hongru, Burke Chris, Kwiatkowski Thomas J., Sapp Peter, McKenna-Yasek Diane, Brown Robert H., Hayward Lawrence J. (2010). Mutant FUS proteins that cause amyotrophic lateral sclerosis incorporate into stress granules. Human Molecular Genetics.

