# Supplementary material for: Segregation and potential functional impact of a rare stop-gain PABPC4L variant in familial atypical Parkinsonism
Source: Sci Rep. 2019 Sep 19;9:13576. doi: 10.1038/s41598-019-50102-6 (PMC6753086; doi:10.1038/s41598-019-50102-6)
Supplement: Supplementary file 1 — Supplementary file [file 41598_2019_50102_MOESM1_ESM.pdf]

Segregation and potential functional impact of a rare stop-gain PABPC4L variant in familial atypical Parkinsonism

Muhammad Aslam[a], Anwar Ullah[a,b,c], Nagarajan Paramasivam[d], Nirosiya Kandasamy[a], Saima Naureen[a,e], Mazhar Badshah[f], Kafaitullah Khan[g], Muhammad Wajid[h], Rashda Abbasi[b], Roland Eils[i,j], Marc A. Brockmann[k], Matthias Schlesner[l], Nafees Ahmad[b], Jakob von Engelhardt[a]

Supplementary table: Genotyping by Sanger sequencing of the three candidate causative variants located in three different genes to validate the disease cosegregation

**Gene**		**PABPC4L**	**ZNF292**	**C6orf163**
Ensemble transcript ID		ENST00000421491.3	ENST00000369577.3	ENST00000388923.4
NCBI refernce sequence ID		NM_001114734.2	NM_001351444	NM_001010868
UniProd ID		P0CB38	O60281	Q5TEZ5
Variation (cDNA; Protein)		SNV (c.C811T:p.R271X)	SNV (c.G4918C:p.A1640P)	SNV (c.C215T:p.A72V)
GRCh37 Chrom_pos_ref_alt		4_135121364_G_A	6_87968265_G_C	6_88058596_C_T
**Status**	**Pedigree ID**	**Genotyping by Sanger sequencing**		
**Affected**	III:1*	C/T	G/C	C/T
	III:3	C/T	G/C	C/T
	IV:4	C/T	G/G	C/T
	IV:7	C/T	G/G	C/C
	IV:9	C/T	G/G	C/C
	IV:11*	C/T	G/C	C/T
**Unaffected**	III:2*	C/C	G/G	C/C
	III:6	C/C	G/C	C/T
	III:7	C/C	G/G	C/T
	III:8	C/C	G/G	C/C
	III:12	C/C	G/G	C/C
	III:13	C/C	G/G	C/C
	IV:1	C/C	G/G	C/C
	IV:3	C/C	G/C	C/C
	IV:5	C/C	G/C	C/C
	IV:6	C/C	G/G	C/T
	IV:8	C/C	G/G	C/C
	IV:10	C/C	G/C	C/C
	IV:12*	C/C	G/G	C/C

*Individuals for whom WGS has been performed

Supplementary figure S1: Full blots for Extended Data Figure 2c

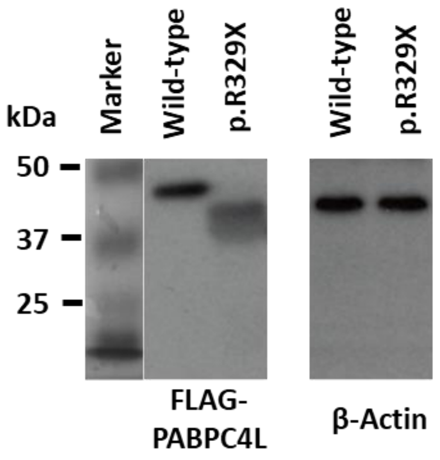

Supplementary figure S2: Heatmap representation of transcript expression profiles of the four type I cytoplasmic PABP family members in adult human brain (image credit: Human microarray data from the Allen Brain Atlas).

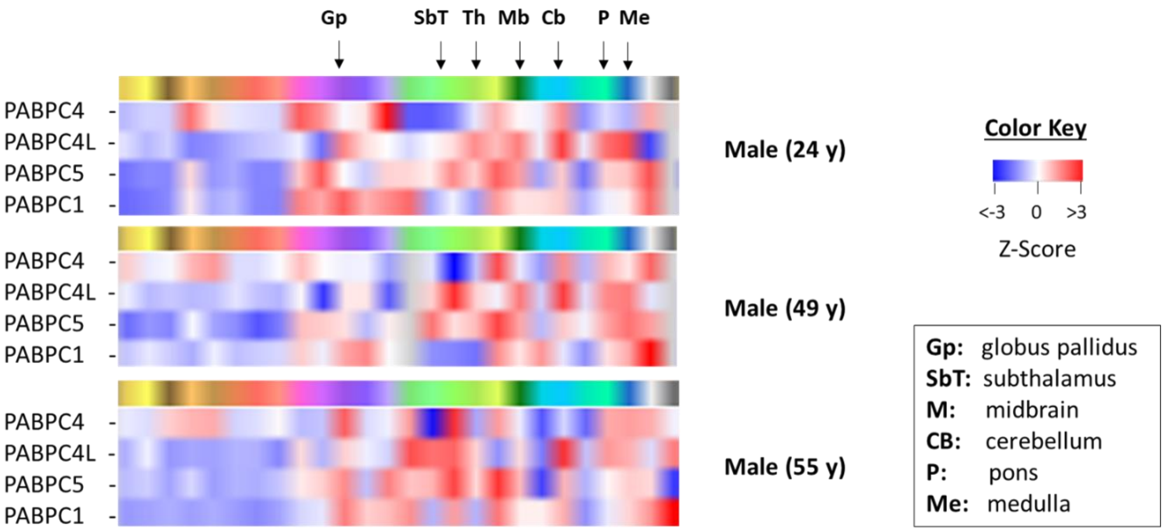

Heatmaps showing normalized Z-score in "Coarse" mode representing transcript expression profiles of the type I cytoplasmic PABPs in different brain regions (multi-color ribbon). Regions with detectable PABPC4L transcript expression are highlighted. Expression data from three different donors is shown covering the age of onset of clinical symptoms in the investigated family.

Image credit: Allen Institute https://human.brain-map.org/microarray/search/show?search_type=user_selections&user_selection_mode=1.
